# Protective mechanism of quercetin and its derivatives in viral-induced respiratory illnesses

**DOI:** 10.1186/s43168-022-00162-6

**Published:** 2022-11-18

**Authors:** Wahyu Choirur Rizky, Muhammad Candragupta Jihwaprani, Mazhar Mushtaq

**Affiliations:** 1 College of Medicine, Sulaiman Al Rajhi University, Al Qassim, Saudi Arabia; 2 Department of Basic Sciences, College of Medicine, Sulaiman Al Rajhi University, Al Qassim, Saudi Arabia

**Keywords:** Antiviral, Quercetin, Respiratory tract infections, Viral infections

## Abstract

Globally, acute respiratory illnesses are the most commonly manifesting illness in all age group. The disease mostly affects the upper respiratory tract (URT) and is self-limiting. However, a small percentage progresses to lower respiratory tract infections (LRTI). The most important causative agents of severe LRTIs are bacteria and viruses. Various viruses can cause respiratory tract infections, being the most essential belonging to the Orthomyxoviridae, Paramyxoviridae, Picornaviridae, coronaviruses, and adenoviruses. Quercetin is classified as a flavonoid compound and was previously known to have antiviral, antibacterial, antioxidant, and anti-inflammatory activities. Some preclinical studies highlight quercetin could also interfere with coronavirus infection and modulate the release of pro-inflammatory cytokines. Since there is no comprehensive compilation addressing the antiviral activities of quercetin and its derivatives, this narrative review provides a summary of the preclinical evidence of their antiviral activities on respiratory illnesses induced by viruses other than coronaviruses. The literature research was performed in PubMed, Scopus, and Google Scholar. The results explain that quercetin has a wide range of actions in viral-induced respiratory illnesses including, but not limited to suppressing pro-inflammatory cytokines and chemokines, promoting antioxidant-related genes expression, blocking viral entry and replication, accelerating viral clearance, reducing the accumulation of alveolar macrophages, and reducing goblet cells marker and mucin gene expression.

## Background

Worldwide URT illnesses are the most commonly manifesting illness in all age groups. Notwithstanding, a small percentage progresses to LRTI, like bronchiolitis and pneumonia. In developing countries, pediatric and elderly populations are at increased risk of contracting the disease [1]. The most important etiologic agents of severe LRTI are bacteria like *Streptococcus pneumoniae* and *Haemophilus influenzae* and viruses like influenza virus and respiratory syncytial virus (RSV). Bacteria are the main cause of pneumonia in adults, whereas viruses are the main cause of mild upper and middle respiratory tract infections (RTIs) and in bronchiolitis pediatric [1].

Various viruses can cause RTIs, being the most essential belonging to the Orthomyxoviridae, Paramyxoviridae, Picornaviridae, coronaviruses, and adenoviruses. Albeit the majority of mild RTIs are caused by virus, recent systemic review and meta-analyses reported that in adult populations, 25% of patients diagnosed with community-acquired pneumonia was also known to be virus in origin. There was a strong causal association with RSV, influenza viruses, parainfluenza viruses, and metapneumovirus in children under 5 diagnosed with LRTIs as compared to healthy controls. Whereas in adults, some viruses frequently contributed to LRTIs such as influenza viruses 8%, rhinoviruses 6%, coronaviruses 3%, and RSV 2% [2, 3].

Quercetin is a plant pigment and is classified as a flavonoid compound that has a polyphenol or pentahydroxyflavone structure (C_15_H_10_O_7_) [4]. Naturally, it is ubiquitous in plant food sources (primarily as glycosides) like onions, apples, citrus fruits, green leafy vegetables, kale, broccoli grapes, cherries, berries, buckwheat, and green tea. Among them, onions and apples are the most important sources of quercetin in the human diet. Likewise, it has been considered a major bioflavonoid compound in the human diet and renders several benefits to human health [5]. In contrast to naturally occurring quercetin which is mainly found in a form of glycoside, dietary supplements in the marketplace which contain quercetin are provided as a free form of quercetin, which is the aglycone.

Pharmacologically, quercetin has been evaluated for its antiviral and antibacterial activity, anti-inflammatory, antiplatelets, antihypertensive, antitumor, neuroprotective effect, cardioprotective effect, gastroprotective effect, natural antihistamine, hepatoprotective, and antiprotozoal [4, 5]. In our previous review, quercetin showed promising molecular effects against novel severe acute respiratory syndrome coronavirus-2 (SARS-CoV-2) through specific mechanisms by blocking the interaction of spike protein (S-protein) on angiotensin-converting enzyme 2 (ACE2) receptor, effects on 3C-like protease (3CL^pro^), papain-like protease (PL^pro^), and RNA-dependent RNA polymerase (RdRp) [6]. Moreover, in some clinical trials, quercetin has shown a promising effect on clinical improvement in respiratory symptoms in COVID-19 subjects [4, 7]. Several studies have shown that quercetin and its derivatives demonstrated promising effects on various lung-induced injury models including the fibrotic lung model and acute lung injury in the sepsis model [6, 8]. Notwithstanding, the evidence of the antiviral activity of quercetin and its derivatives against viral-induced respiratory infections is still lacking. Hence, our literature review aims to discuss the evidence of antiviral and anti-inflammatory properties of quercetin in molecular and preclinical studies specifically on viral agents that are responsible for respiratory infections.

## Methods

The literature search was performed in PubMed, Scopus, and Google Scholar using the Medical Subject Heading (MeSH) term “Quercetin” combined with the following descriptors: “Virus,” “Viruses,” “Respiratory Tract,” “Pulmonary,” and “Infections.” These descriptors were connected using the connector “AND” between them as follows: “Quercetin” AND “Virus” AND “Respiratory Tract Infections.” Only studies written in English were chosen, and the full papers were selected from 2010 to 2022.

### Quercetin abrogates the respiratory illnesses induced by rhinovirus

Beyond the promising evidence of quercetin activities in modulating inflammatory responses and oxidative damages in ALI-induced mice models, there were some studies supporting the antiviral effect of quercetin in lower respiratory tract-viral-induced models. In vivo study conducted by Farazuddin et al. revealed that quercetin has the ability to significantly alleviate rhinovirus (RV)-induced inflammatory alterations and the progression of lung illness in a mouse model of COPD [9]. The results showed that mice on quercetin supplementation demonstrated 1 to 2 log lesser viral RNA at all time points as compared to mice with a control diet. Sustained elevation of inflammatory cytokines was noted during prolonged RV infection both in normal mice and COPD-phenotype mice. Intriguingly, there was no elevation of the protein or mRNA levels of chemokine (C-C motif) ligand 3, chemokine (C-X-C motif) ligand 1 (CXCL-1), CXCL-10, IFN-γ, IL-17, and TNF-α in mice receiving quercetin supplementation. Moreover, concomitant RV infection in mice with COPD phenotype induces a further elevation in T cells, neutrophils, and macrophages. Again, quercetin supplementation consistently showed promising effects to reduce the level of these three cells corroborating with declining chemokine levels [9]. In contrast to the normal group, COPD-phenotype mice demonstrated a small elevation in the population of alveolar and intermediate macrophages, and following RV infection, there was a pronounced elevation of intermediate macrophages. The elevation of these two macrophages population and lung monocyte populations in RV-infected mice with COPD was significantly abrogated by quercetin supplementation. Another substantial finding of this study was that quercetin remarkably abated RV-induced inflammatory changes and progression of pulmonary disease in COPD mice, demonstrated by a decrease in goblet cell number and expression of goblet cell markers (Gob5) and mucin gene. These effects were due to the powerful antioxidant and anti-inflammatory of quercetin, which reduces the prolonged activation of epithelial cells generated by RV, hence reducing immune cell buildup and activation. It is conceivable that quercetin may mitigate RV-induced pathogenic effects not only by suppressing the host inflammatory responses to RV but also by reinforcing the viral clearance [9].

**Fig. 1:**
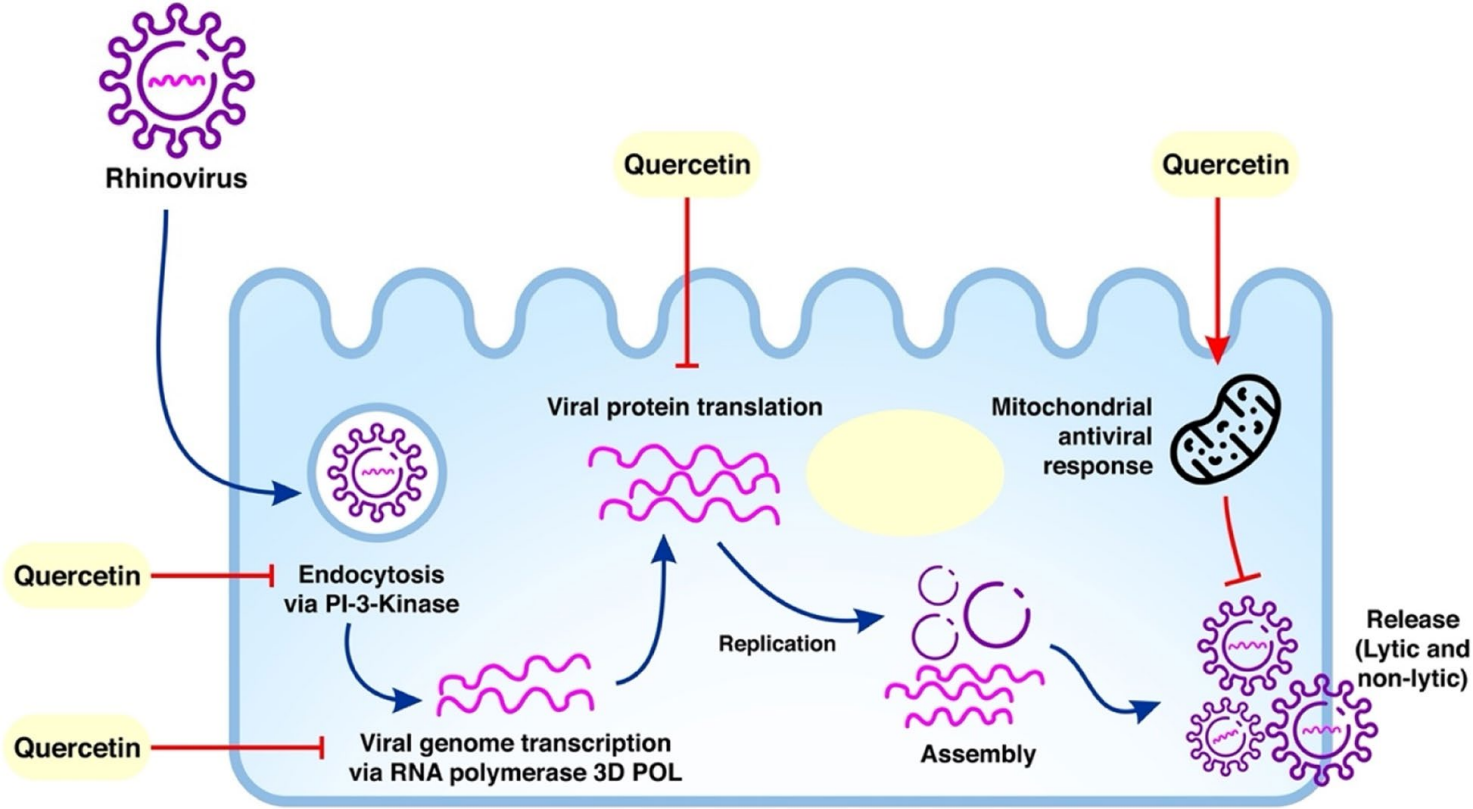
Inhibition of rhinovirus replication at various stages by quercetin. Quercetin blocks viral endocytosis via inhibition of PI3k/Akt pathway, viral genome transcription by inhibiting RNA polymerase 3D POL, and viral protein translation by facilitating eIF4G cleavage. Likewise, quercetin enhances viral clearance via mitochondrial antiviral response [10]

Another study regarding the effect of quercetin against RV infection was explored by Ganesan et al.; it reveals that quercetin supplementation before or during RV infection has the ability to diminish RV infection at multiple stages in the viral life cycle [10]. In cultured airway epithelial cells, pretreatment with 10 μM quercetin impedes both RV- and UV-irradiated-RV-induced IL-8 responses by 75–80%. The concentration required to inhibit viral replication was higher than in vivo, suggesting that quercetin achieves better absorption in vivo, thus rendering better stability and bioavailability. Another possibility is that quercetin metabolites generated in vivo act stronger than those in vitro. The result showed that quercetin pretreatment suppressed RV-induced Akt phosphorylation. Inhibition of RV endocytosis and PI3k/Akt signaling by quercetin is stipulated as key factors to reduce IL-8 expression (Fig. 1). When quercetin was given after viral endocytosis, it reduced RV-stimulated IL-8 and IFN responses, which was consistent with downstream effects. Quercetin hindered RV replication by inhibiting RV genome transcription. Quercetin, on the other hand, inhibited RV-induced cleavage of eIF4GI while increasing the phosphorylation of eIF2a [10]. Because quercetin promotes eIF2a phosphorylation beyond that triggered by RV infection, there is a possibility that quercetin restricts viral replication by boosting host innate immune responses. Recently, it is shown that replication of enterovirus, a Picornaviridae family member, takes place in specialized organelles rich in phospho-inositide-4-phosphate (PI4P) lipids produced by PI-4-kinase IIIβ. If PI4P-enriched organelles are necessary for RV reproduction, quercetin, which inhibits PI-4-kinases, may interfere with the development of these organelles, and ultimately, the viral replication is inhibited. The results further showed that cleavage of eIFG4II and viral capsid protein levels were significantly lowered by quercetin, thus eventually decreasing positive- and negative-strand viral RNA. This mechanism implies that quercetin may inhibit initial polypeptide processing, which is required for both viral RNA polymerase processing and cleavage of eIFG4II, thereby blocking all downstream effects. Another probability is that quercetin inhibits viral RNA polymerase directly, preventing genome translation and the manufacture of new offspring viruses [10].

### Quercetin abrogates the respiratory illnesses induced by influenza virus

**Fig. 2:**
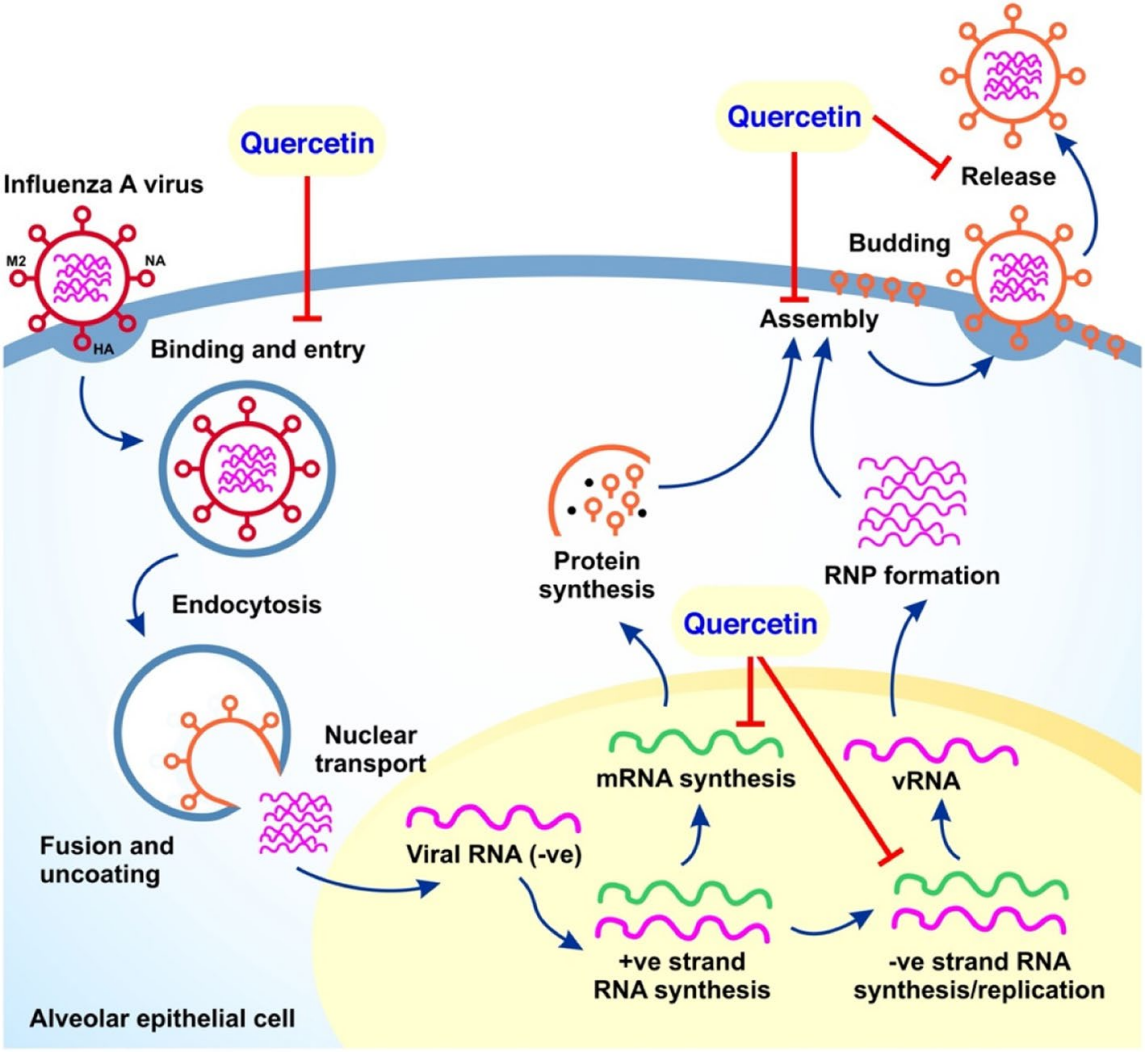
Illustration of influenza virus life cycle and the pathways modulated by quercetin. Influenza virus utilizes hemagglutinin (HA) receptor for binding and entry. Moreover, membrane-2 (M2) ion channel promotes fusion and uncoating, and neuraminidase (NA) facilitates the progeny virus release. Collectively, quercetin interferes at different steps including viral binding, uncoating, mRNA synthesis, negative-strand synthesis, assembly, and release [12]

Oral treatment with quercetin 3-rhamnoside (Q3R) as compared to placebo and oseltamivir-treated groups showed effectiveness against influenza A/WS/33 (H1N1) virus in mouse models [11]. The survival rate is significantly increased, on average 100%, by administration of Q3R and oseltamivir in contrast to placebo-treated control mice. Typical influenza lesions including interstitial pneumonia and necrotizing bronchiolitis were also noticed. Q3R administration on influenza-infected mice showed moderate inflammation including pulmonary edema and elevated numbers of inflammatory cells and necrosis. Particularly, a dose of 6.25 mg/kg of Q3R hampered the progression and development of pulmonary lesions and postinfection pulmonary edema as compared to oseltamivir-treated mice; suggesting Q3R greatly reduces adverse outcomes and influenza-related severity. Moreover, the result of the CPE_50_ assay to determine the viral titer in the mice lung demonstrated the average titer for Q3R-treated mice was approximately 2000 lower than that of the placebo group and two times lower than that of the oseltamivir-treated group. Intriguingly, Q3R and oseltamivir treatment given for influenza uninfected mice displayed similar histopathological results to placebo-controlled mice [11]. The influenza virus envelope protein hemagglutinin has been targeted to block the viral entry by pretreatment and co-treatment with quercetin [12]. It revealed that quercetin generated an obvious inhibitory action against H3N2 and H1N1 virus strain infections in a dose-dependent manner. The inhibitory effect was pronounced when the virus was preincubated with quercetin or when quercetin was added to the virus-infected cells (MDCK and A549 cells). Based on the time of additional assay, quercetin effectively acts during the viral entry stage, while inhibitory effects during or after infection were less clear. Furthermore, the results confirm that quercetin generated a strong binding affinity to the HA2 subunit of influenza A virus (Fig. 2) which is responsible for the fusion of the viral envelope with the endosomal membrane [12].

There has been some evidence during acute influenza virus infection that oxidative stress plays a crucial role in the living cells affected [13]. This process eventually promotes lung injury, apoptosis, inflammation, or allergy and further alters cellular metabolism, particularly mitochondrial function resulting in mitochondrial ROS production [13, 14]. Instillation of influenza virus A/Udorn/317/72(H3N2) in mice model intranasally significantly reduced the pulmonary concentrations of SOD and reduced glutathione (GSH/GSSG), CAT, and vitamin E [12]. Oral administration of quercetin 1 mg per day following the viral instillation demonstrated a positive effect to elevate the level of SOD, the ratio of GSH/GSSG, and CAT in the lung homogenate. However, quercetin supplementation failed to increase the vitamin E level significantly in both normal and infected-H3N2 mice models. The study concluded that quercetin proved to raise the antioxidant concentration in the lung and bring them nearly to normal levels. These findings consistently supported that quercetin demonstrated the ability to block ROS production by neutrophils, particularly by blocking protein kinase C (PKC), an enzyme that activates NADPH oxidase and respiratory burst [15]. A previous in vitro study by Wan et al. showed that quercetin has the ability to reduce the mRNA and protein overexpression of cyclin-dependent kinase-4 (CDK4) in A459 lung epithelial tumor cells infected with H1N1 [16]. At a minimum effective concentration of 10 mg/l, the addition of quercetin in the infected cells model caused inhibition of the H1N1 cytopathic effect. The expression of CDK4 mRNA and protein in H1N1-infected cells treated with quercetin and ribavirin was significantly lower than in untreated infected cells. Albeit the direct antiviral action of quercetin on H1N1 was not as strong as ribavirin, the result showed quercetin was less toxic and able to inhibit CDK4 mRNA and protein overexpression in H1N1-infected A459 cells. These findings are presumably due to the ability of quercetin to induce DNA repair, thus promoting host cell proliferation [16].

Another study by Gansukh et al. [17] performed the isolation of quercetin-7-O-glucoside (Q7G) from *Dianthus superbus* by bioassay (anti-influenza)-guided fractionation. The antiviral activity of Q7G was evaluated against influenza A/Vic/3/75 (H3N2), A/PR/8/34 (H1N1), B/Maryland/1/59, and B/Lee/40 viruses. Likewise, virus-induced ROS and autophagy formation were also evaluated. The result showed that Q7G was considered not to be cytotoxic (CC_50_ > 100 μg/ml) in MDCK cells. The IC_50_ values against influenza A/Vic/3/75 (H3N2), A/PR/8/34 (H1N1), B/Maryland/1/59, and B/Lee/40 virus strains were 6.61 μg/ml, 3.1 μg/ml, 5.17 μg/ml, and 8.19 μg/ml, respectively.

### Acetylated quercetin blocks the human respiratory syncytial virus (hRSV) entry, adhesion, and replication

Human respiratory syncytial virus (hRSV), a member of the family Pneumoviridae, is an important pathogen in the development of acute lower respiratory infection (ALRI) and a major cause of hospitalization in the pediatric population worldwide. To date, the existing treatment and management of hRSV infections like the use of palivizumab and ribavirin encounter challenges. For instance, difficulty in administration, the generation of drug escape mutants, substantial side effects, long-term administration, low efficacy against the virus, and expensive cost are entailed in prophylaxis. Hence, some researchers have been done to discover alternative compounds or design new strategies against hRSV to suppress its spread and halt the infection [18].

Quercetin is a bioactive flavonoid that confers several biological effects, including anti-hRSV [19]. Notwithstanding, the issue still exists pertaining to low solubility and low stability of quercetin in the lipophilic media of a membrane owing to the presence of hydroxyl groups [20]. A previous study done by Lopes et al. used quercetin as a parent compound (Q0) and acetylation of quercetin; quercetin pentaacetate (Q1) was tested on Hep-2 cells infected with hRSV to evaluate its capability in halting the viral entry and replication [21]. Initially, cytotoxicity of Q0 and Q1 was done in incubated Hep-2 cells in the selected range of concentration 0.5–1,024 μM for 3 days, and the cells viability was verified by MTT assays. The cytotoxicity concentration 50% (CC_50_) values for Q0 and Q1 were 11 and 37 μM, respectively. The results revealed that the Q1 had lower cytotoxicity than the Q0. However, post-treatment using both compounds and testing of virucidal activity on the hRSV-infected cell were done using three different multiplicities of infection (*MOI* = 0.1, 0.5, and 1.0). As compared to Q0, Q1 showed relevant cell protection (2.5–10 μM) in post-treatment at the lowest MOI and specifically conferred excellent anti-hRSV virucidal effect (90–95% protection on the Hep-2 cells) at the lowest MOI by the concentration of 10 μM. Likewise, in a dose-dependent manner, the infected cells treated with Q1 revealed a reduction of syncytia formation, no cell detachment in post-treatment at the lowest MOI, and a great reduction of syncytia formation in the virucidal assay at MOI 0.1 and 0.5 [21]. Concerning cytotoxicity, it has been shown that Q1 is more hydrophobic than Q0. Hence, Q1 would penetrate cells by which it is protected by degradation of extracellular space [22].

To further understand, in silico analysis was done in the same study to prove that the compound may interact with the F-protein of hRSV [21]. Similar to G, the F-protein has been established to interact with cellular heparan sulfate or immobilized heparin, facilitating attachment to and infection of immortalized cells [18]. The study verified the effect of Q1 on the stability of the hRSV-F modeled (hRSVmF)-Q1 complex and used the hRSVmF-JNJ-2408068 as reference control. The docking results showed that Q0, Q1, and the reference ligand completely occupy the hRSVmF central cavity and exhibit similar interaction energies (−6.5 kcal/mol). Q0 demonstrated an escaping trend from the hRSVmF central cavity, which did not happen with the reference ligand. Intriguingly, Q1 exhibited a closer interaction with the cavity when compared to Q0 and towards the end of the trajectory demonstrated a similar pattern to the reference ligand. There were no hydrogen bonds observed between Q1 and hRSVmF cavity residues, showing the occurrence of hydrophobic interactions. The hRSVmF-Q1 complex revealed higher van der Waals energy (−36.0 kcal/mol) and lower electrostatic interaction (−21.1 kcal/mol) as compared to Q0 (−18.2 kcal/mol; −32.0 kcal/ mol respectively). Albeit Q1 exhibited lower electrostatic contributions and did not exert hydrogen bonds with hRSVmF cavity residues, the interaction energy of Q1 is higher than that observed for Q0, indicating the structural differences between the two molecules. The chemical modification from Q0 to Q1 implies the replacement of the hydroxyl group for acetyl, hence resulting in greater affinity for the cavity hRSVmF [21].

The hRSV cycle entails adsorption, internalization, transcription, translation, assembling, and budding. Previous studies revealed that the hRSV infection cycle is complete in about 24–48 h postinfection (hpi) [23]. According to the same study, the effect of Q1 in the hRSV cycle was tested in adsorption, internalization, and time addition protocols with *MOI* 0.5, and the viability of HEp-2 monolayer was observed 3-day postinfection (dpi) by MTT assay. The result showed that Q1 exhibits great protection on adsorption assay in all tested concentrations (1.2–10 μM). Notwithstanding, in internalization protocol, Q1 did not exhibit any effect on hRSV infection. Moreover, Q1 at 6 μM only exhibited significant protection in time addition protocol in 0 and 36 hpi [21].

Another important target protein possessed by hRSV is the M2-1 protein. It is an essential anti-termination factor for the transcription process that averts the premature dissociation of the polymerase complex. Hence, it becomes a potential target for the development of viral replication inhibitors [24]. A study done by Guimaraes et al. evaluated the interaction of Q1 and tetraacetylated (Q2) quercetin derivatives with the M2-1 tetramer. The acetylation was done to generate a stronger bioactive compound against the oxidation process. Based on fluorescence spectroscopy results, it has been shown that the binding constants of M2-1/compounds complexes were present on the order of 10^4^ M^−1^, given that the Q2 binding affinity was stronger than Q1. Likewise, Q2 exhibited more hydrogen bonds than Q1 indicated by higher *K*_b_ values of the M2-1/Q2 complex. Furthermore, the thermodynamic analysis revealed that hydrophobic interaction possesses an important role in the formation of M2-1/compound complexes. To further understand the formed interaction, molecular docking was done, and it demonstrated that the possible binding site takes place between the globular domain (α-helix 6) from one monomer with the zinc finger domain from other monomers of the tetramer. The hydrogen bonds and stacking interactions are known to promote the stabilization of the M2-1/compound complexes [24]. Providing these results, acetylated quercetin renders its potential as an hRSV inhibitor since it was able to generate binding sites in the viral RNA-binding domain.

### Quercetin inhibits cytokines release and suppresses viral load in Human metapneumovirus infection

*Human metapneumovirus* (hMPV) is known as the major cause of lower respiratory tract infections, for instance, bronchiolitis and pneumonia in pediatrics, elderly, and immunocompromised individuals. Notwithstanding, the economic impact on the pediatric population is similar to that of the influenza virus [25]. To date, hMPV infection of airway epithelial cells stimulates pro-inflammatory gene expression via activation of transcription factor nuclear factor kappa B (NF-κB) p65 subunit and interferon regulatory factor (IRF)-3. Likewise, both in vitro and in vivo, it stimulates oxidative stress by progressively deteriorating gene expression and protein levels of antioxidant enzymes like superoxide dismutase-3, catalase, and glutathione S-transferase. The nuclear translocation of the transcription factor Nrf2 was also known to be reduced in hMPV infection [26].

An in vitro study was done using A549 cells (alveolar type 2 cancerous cell line) that were infected with hMPV at an MOI of 1 [25]. Following 24-h postinfection, there was evidence of an increased level of 8-isoprostane (oxidative stress damage marker), CXCL8 (IL-8), CCL5 (RANTES), IL-1α, IL-6, TNF-α, CXCL10 (IP-10), and CCL4 (MIP-1β). Treatment with both resveratrol (10–50 μM) and quercetin (1–10 μM) in a dose-dependent manner was significantly able to suppress the expression of these cytokines and chemokines [25]. Moreover, treatment with resveratrol and quercetin given 24-h post-infection was associated with a much lower viral titer, as compared to control (untreated but infected by hMPV). In the resveratrol group, the viral titer declined from almost a million per mL to 50,000, and in quercetin group, the viral titer declined to a little above 100,000 [25]. This study also investigated whether resveratrol and quercetin could suppress the activation of pro-inflammatory cytokines NF-κB and IRF-3 activation. The result demonstrated that hMPV infection caused significant p65 subunit and IRF-3 nuclear translocation. hMPV infection drastically increased NF-κB/p65 binding to the IL-8 promoter and IRF-3 binding to the RANTES promoter. Likewise, treatment with resveratrol and quercetin significantly reduced these actions, thus suppressing the subsequent release of pro-inflammatory cytokines and chemokines [25].

## Conclusion

All these studies explained how quercetin and its derivatives demonstrate a wide spectrum of antiviral activities on respiratory illnesses induced by various viral infections. Better comprehension of mechanistic and pharmacokinetics properties of quercetin could help in the rational design of more bioavailable and/or potent flavonol-type compounds. Albeit many in vitro studies exist, clinical studies using human subjects are still lacking. It would be of extreme importance to focus on the utilization of quercetin for prophylaxis or preventive purposes, as well as in combination with other medications. Hence, it could enhance or promote synergistic interactions between the substances and reduce the adverse effects and related toxicity and increase the overall safety and efficacy.

## Data Availability

Not applicable

## Abbreviations

URT: Upper respiratory tract
LRTI: Lower respiratory tract infections
RSV: Respiratory syncytial virus
RTI: Respiratory tract infections
ACE2: Angiotensin-converting enzyme 2
SARS-CoV-2: Severe acute respiratory syndrome coronavirus-2
S-protein: Spike protein
3CLpro: 3C-like protease
PLpro: Papain-like protease
RdRp: RNA-dependent RNA polymerase
RV: Rhinovirus
C-C motif: Chemokine
CXCL-1: Chemokine (C-X-C motif) ligand 1
Gob5: Goblet cell markers
PI4P: Phospho-inositide-4-phosphate
Q3R: Quercetin 3-rhamnoside
GSH/GSSG: Reduced glutathione
PKC: Protein kinase C
CDK4: Cyclin-dependent kinase-4
Q7G: Quercetin-7-O-glucoside
hRSV: Human respiratory syncytial virus
Q0: Quercetin as a parent compound
Q1: Quercetin pentaacetate
Q2: Quercetin tetraacetylate
hMPV: Human metapneumovirus
NF-κB: Nuclear factor kappa B
IRF: Interferon regulatory factor
